# Metastatic bladder cancer forming a sigmoidorectal fistula after enfortumab vedotin therapy: a case report

**DOI:** 10.3389/fonc.2023.1274494

**Published:** 2023-11-09

**Authors:** Shinji Tamada, Daiki Ikarashi, Naoki Yanagawa, Moe Toyoshima, Kenta Takahashi, Tomohiko Matsuura, Shigekatsu Maekawa, Renpei Kato, Mitsugu Kanehira, Ryo Takata, Wataru Obara

**Affiliations:** ^1^ Department of Urology, Iwate Medical University School of Medicine, Iwate, Japan; ^2^ Department of Pathology, Iwate Medical University School of Medicine, Iwate, Japan

**Keywords:** enfortumab vedotin, sigmoidorectal fistula, bladder cancer, nectin-4, comprehensive genomic profiling

## Abstract

We report the case of a 68-year-old man who developed a sigmoidorectal fistula after marked response to enfortumab vedotin for advanced bladder cancer. The patient had undergone radical cystectomy with ileal conduit after neoadjuvant chemotherapy. Six months after surgery, local recurrence in the pelvic cavity and multiple lung metastases were found, and the patient was administered pembrolizumab as second-line therapy. Due to worsening local recurrence and suspected invasion of the sigmoid colon and rectum, enfortumab vedotin was initiated as third-line therapy and comprehensive genomic profiling was simultaneously performed. Enfortumab vedotin was remarkably effective, the lung metastases disappeared, and the local recurrent lesion shrank in volume although a sigmoidorectal fistula was found to form through the tumor cavity. Immunohistochemical analysis of the tumor specimens exhibited increased nectin-4 expression. This rare case of metastatic bladder cancer with sigmoidorectal fistula associated with effective enfortumab vedotin therapy suggests that nectin-4 expression and comprehensive genomic profiling might be useful in predicting treatment response to enfortumab vedotin.

## 1 Introduction

Enfortumab vedotin (EV) is a novel antibody–drug conjugate that includes a human anti-nectin-4 antibody conjugated to the highly potent microtubule-disrupting agent monomethyl auristatin E (MMAE) (1). The efficacy of EV therapy for locally advanced or metastatic urothelial carcinoma (UC) was demonstrated in the EV-301 trial, which included patients who experienced disease progression during or after treatment with a programmed death-1 or programmed death-ligand 1 (PD-L1) inhibitor following prior treatment with platinum-based chemotherapy (2). Although EV therapy is associated with overall survival benefit, the response rate about tumor shrinking effect is limited.

EV targets the transmembrane form of nectin-4. In the EV-101 study (3), almost all tumors, including metastatic UC, exhibited strong nectin-4 expression, which led to a protocol amendment to omit the assessment of nectin-4 expression before enrollment in that pivotal trial. However, nectin-4 expression has been reported to exhibit heterogeneity between the primary and metastatic sites and between histologic UC subtypes (4).

Sigmoidorectal fistula is a clinical presentation sometimes observed in patients with cancer or inflammatory disease, such as colorectal cancer or diverticulitis (5, 6). Although one study reported the development of gastrocolic fistula after targeted therapy for gastric cancer (7), to our knowledge no study to date has reported sigmoidorectal fistula due to EV in patients with cancer.

Here, we report the case of a patient with bladder cancer who exhibited a dramatic response to EV therapy but developed sigmoidorectal fistula through the tumor cavity; immunohistochemical analysis revealed increased nectin-4 expression in the tumor.

## 2 Case description

A 68-year-old man with the chief compliant of macrohematuria presented to our hospital. His medical history included angina pectoris and paroxysmal atrial fibrillation. Extensive investigation led to the diagnosis of locally advanced bladder cancer (cT3N0M0). After three cycles of gemcitabine plus cisplatin as neoadjuvant chemotherapy, the patient underwent robot-assisted radical cystectomy with ileal conduit. The postoperative pathologic evaluation led to the diagnosis of high-grade (G2/G3) UC, pT4apN0, with negative surgical margin. Evaluation at six months after surgery revealed multiple pulmonary metastases and a local recurrent lesion in the pelvic cavity. Despite the initiation of pembrolizumab as second-line chemotherapy, these lesions were found to be aggressively enlarged at the end of 10 cycles of pembrolizumab. Therefore, EV was considered as third-line therapy.

**Figure 1:**
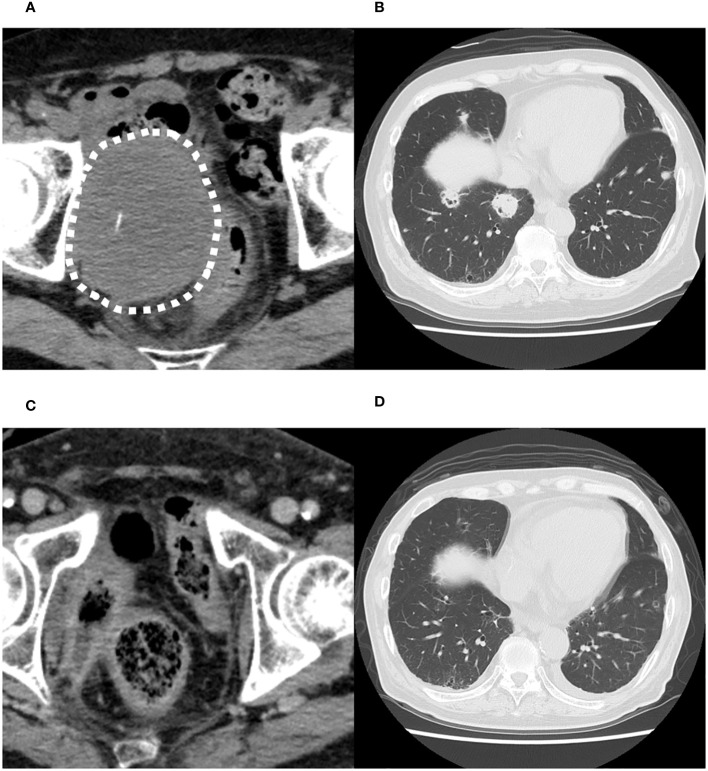
Computed tomography images before the initiation of enfortumab vedotin therapy. **(A)** Recurrent tumor in the pelvic cavity suspected to invade the sigmoid colon and rectum. **(B)** Multiple metastases in both lungs. Computed tomography images after the second course of enfortumab vedotin therapy. **(C)** Recurrent tumor in pelvic cavity has dramatically shrunk. **(D)** Multiple lung metastases have disappeared.

**Figure 2:**
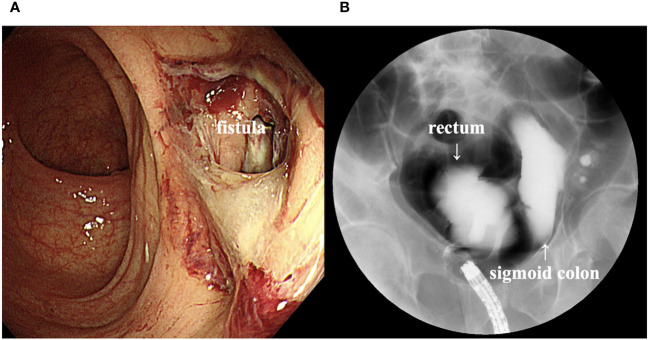
**(A)** Colonoscopy image showing a fistula in the lateral rectal wall. **(B)** Contrast enema image showing inflow of the contrast agent into the sigmoid colon through the rectum.

Computed tomography (CT) scan obtained before EV initiation revealed a recurrent tumor invading the sigmoid colon and rectum and multiple lung metastases in bilateral lungs (**Figures 1A, B**). The patient had the subjective symptom of narrowing of defecation, and the creation of a colostomy was considered based on discussion with a surgeon. Given that the patient was able to defecate and did not exhibit ileus, we decided not to create a stoma at this point. We decided to follow up the patient with imaging evaluation at short intervals, and to create colostomy immediately if ileus symptoms appeared. EV was initiated at a dose of 1.25 mg/kg. The patient developed grade 1 skin rash according to the Common Terminology Criteria for Adverse Events on day 15 of the first course. On day 15 of the second course, skin rush worsened to grade 3 and grade 2 anorexia also appeared. Therefore, the patient was referred to a dermatologist and oral and topical steroids were initiated, which led to improvement in skin rash. The EV dose was reduced, starting in the next cycle. At that time, the patient experienced an episode of white stool. Worsening of disease was considered, and comprehensive genomic profiling (CGP) of a cystectomy specimen collected at the time of EV initiation was performed using FoundationOne^®^ CDx (Foundatione Medicine, USA) (**Table 1**). CT scan after the second EV course revealed the disappearance of multiple lung metastases and dramatic shrinkage of the recurrent tumor in the pelvic cavity (**Figures 1C, D**). However, a fistula between the sigmoid colon and rectum through the tumor cavity was suspected, which was considered the result of the reduction in tumor size. Colonoscopy revealed that the fistula was in the lateral rectal wall (**Figure 2A**). Furthermore, contrast enema revealed the inflow of the contrast agent into the sigmoid colon through the rectum, confirming the formation of a fistula through the tumor cavity (**Figure 2B**). The intestinal wall was not perforated as the fistula was formed through the tumor cavity. At this time, we again suggested the patient to create colostomy. However, the patient did not wish to create colostomy due to decline of his performance status and the difficulty of managing coexistence with a urinary stoma. As a result, the patient was able to continue EV therapy without fatal complications for three months.

**Table 1:** Comprehensive genomic profiling of the patient.

Gene Mutation				
No	Gene	Variant Type	Mutation	Allele Frequency
1	*KDM6A*	Splicing Variant	n/a	0.3633
2	*KRAS*	Missense	p.G12V	0.3786
3	*STAG2*	Nonsense	p.Q963	0.3206
4	*TERT*	Promoter Variant	n/a	0.2128
5	*BRAF*	Missense	p.I326V	0.4513
6	*CD274*	Missense	p.L1425	0.3486
7	*CREBBP*	Missense	p.A1958V	0.569
8	*EP300*	Missense	p.Q275E	0.1164
9	*JAK2*	Missense	p.M1100V	0.3226
10	*MET*	Missense	p.E34D	0.2028
11	*NOTCH1*	Missense	p.R1211W	0.6335
12	*ROS1*	Missense	p.W133R	0.3773
13	*TSC1*	Missense	p.S487C	0.6237
14	*TSC2*	Missense	p.F1574L	0.6523
Copy Number Variation				
No	Gene	Variant type	Copy Number	
15	*CDKN2A*	Deletion	0	
16	*CDKN2B*	Deletion	0	
17	*CRKL*	Amplification	20	
18	*EPHA3*	Amplification	8	
19	*MTAP*	Deletion	0	
Microsatellite Instability				
Stable				
Tumor Mutation Burden				
7 Mutations/Mb				

**Figure 3:**
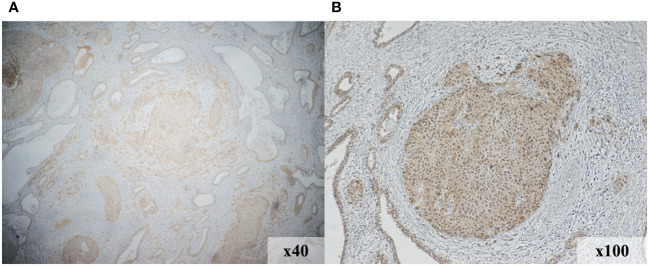
Immunohistochemical staining demonstrates increased nectin-4expression in the tumor region of initial surgical specimens. Magnification, 40× **(A)** and 100× **(B)**.

We investigated the expression levels of nectin-4, CD4, CD8, CD20, and PD-L1 using immunohistochemical analysis of a cystectomy specimens to assess the tumor immune microenvironment. As shown in **Figures 3A, B**, nectin-4 was highly expressed in tumor cells. On the contrary, the surgical specimens included few CD4^+^ and CD8^+^ cells, no CD20^+^ cells, and were negative for PD-L1 expression (**Supplemental Figures 1A–D**).

## 3 Discussion

The present case highlights two important clinical observations, including the formation of a sigmoidorectal fistula during EV therapy in a patient with metastatic bladder cancer and the association of nectin-4 expression and CGP testing with the efficacy of EV therapy.

In the present case, the locally recurrent lesion appeared to enlarge without symptoms, leading to the compression of the rectum and sigmoid colon and the defecation of fecal material with smaller diameter before the introduction of EV therapy. MMAE, the anticancer agent in EV, is a potent microtubule inhibitor and might have caused liquefaction necrosis within the tumor, leading to the formation of the fistula traveling through the tumor cavity to reach the rectum and sigmoid colon. To the best of our knowledge, this is the first report of a sigmoidorectal fistula via tumor cavity following tumor shrinkage. A similar phenomenon was reported in a patient who developed a tracheoparenchymal fistula associated with tumor shrinkage following treatment with docetaxel and bevacizumab (8). In another report, the collapse of tumor cells that directly invade by necrosis or mechanical pressure and the inflammatory peritoneal reaction associated with delayed wound healing were considered as potential mechanisms underlying the formation of a gastrocolic fistula associated with ramucirumab treatment (5). Moreover, in a study comparing ramucirumab monotherapy and combination therapy with ramucirumab and paclitaxel in patients with gastric cancer, the risk for gastrointestinal perforation was slightly higher in those receiving the combination therapy (9). Given that both taxanes and MMAE inhibit microtubules, these observations of fistula formation might be associated with apoptosis of tumor cells. Fortunately, there was no complication including perforation related to sigmoidrectal fistula in this case. Tumor cavity formation may have dispersed intestinal gas pressure. However, perforation could be a potentially fatal complication. Although patient preference of colostomy was not obtained in this case, colostomy creation should be considered for similar cases according to the general condition.

In the present case, the tumor cells exhibited increased nectin-4 expression. Although increased nectin-4 expression is observed in most UC cases, this change is expected to follow a heterogeneous pattern under a variety of circumstances. Miyake et al. reported that nectin-4 expression was decreased in patients who received neoadjuvant chemotherapy (10). Additionally, nectin-4 expression was decreased in metastatic lesions compared to the primary tumor. The recently proposed molecular subtyping of UC has revealed the carcinogenic mechanisms, immune invasion, and clinicopathologic features and is expected to reveal novel biomarkers (11, 12). In recent years, an international consensus has been proposed to categorize UC into six molecular classes: luminal papillary, luminal nonspecified, luminal unstable, stroma-rich, basal/squamous, and neuroendocrine-like (10). This consensus classification holds promise for biomarker discovery research in clinical settings and future clinical trials. Chu et al. (13) reported that the sensitivity of EV was mediated with the expression of nectin-4, which is enriched in luminal subtypes of UC. On the contrary, downregulation of nectin-4 is associated with EV resistance. In the present case, CGP identified the loss of *CDKN2A*/*MTAP* and a mutation in *KDM6A*, suggesting that the UC exhibited the luminal papillary pattern according to the molecular classification. Taken together with these results, we consider that the increased nectin-4 expression and the luminal subtype might be associated with effective EV treatment.

Conversely, our immunohistochemical analyses revealed the lack of PD-L1 expression in tumor cells and the lack of immune cells, including CD4^+^, CD8^+^ and CD20^+^ cells, in the tumor tissue, suggesting that the present case was a cold tumor according to the classification of tumor immune microenvironment. Cold tumors are less immunogenic and less responsive to treatment with immune checkpoint inhibitors (ICIs) (14), which enable the activation and augmented response of cytotoxic T cells. Preexisting tumor-infiltrating lymphocytes in tumor tissue, such as CD4^+^ and CD8^+^ cells, have been reported to be associated with favorable response to ICI therapy in bladder cancer (15). Recent studies reported that tumor-infiltrating CD20^+^ B cells were also associated with survival and immunotherapy response. The prognostic significance of tumor-infiltrating CD20^+^ B cell was generally concordant with that of CD3^+^ and CD8^+^ T cells, and the prognostic effect of T cells was generally stronger in cases where tumor-infiltrating CD20^+^ B cells were also present (16–18). Similarly, Yang et al. (19) reported that bladder cancer could be classified into four subtypes, including cold subtype, based on immune gene profiling. Furthermore, they reported that luminal-type urinary bladder cancers were often cold tumors based on the tumor immune microenvironment. Moreover, the loss of *CDKN2A* and *MTAP* has been reported to attenuate the effect of ICI treatment due to decreased PD-L1 expression and inflammatory or immune cell infiltration (20, 21), consistent with the immunohistochemistry results and the failure of second-line pembrolizumab therapy to control disease in the present case.

In conclusion, the current case of a patient who experienced postoperative local recurrence of bladder cancer, which formed a very rare sigmoidorectal fistula after EV therapy, suggests that we should pay attention to gastrointestinal fistula when we treat the patients whose tumor invade intestinal tract.

## Data availability statement

The original contributions presented in the study are included in the article/**Supplementary Material**. Further inquiries can be directed to the corresponding author.

## Ethics statement

The studies involving humans were approved by Iwate medical university of Medicine institutional review board (IRB 2019-083). The studies were conducted in accordance with the local legislation and institutional requirements. The human samples used in this study were acquired from a by- product of routine care or industry. Written informed consent for participation was not required from the participants or the participants’ legal guardians/next of kin in accordance with the national legislation and institutional requirements. Written informed consent was obtained from the individual(s) for the publication of any potentially identifiable images or data included in this article.

## Author contributions

ST: Conceptualization, Data curation, Formal Analysis, Writing – original draft. DI: Conceptualization, Writing – review & editing. NY: Visualization, Writing – review & editing. MT: Data curation, Writing – review & editing. KT: Data curation, Writing – review & editing. TM: Data curation, Writing – review & editing. SM: Data curation, Writing – review & editing. RK: Data curation, Writing – review & editing. MK: Data curation, Writing – review & editing. RT: Data curation, Writing – review & editing. WO: Conceptualization, Supervision, Writing – review & editing.

## References

[1] Challita-Eid PM Satpayev D Yang P An Z Morrison K Shostak Y. Enfortumab vedotin antibody-drug conjugate targeting nectin-4 is a highly potent therapeutic agent in multiple preclinical cancer models. Cancer Res (2016) 76:3003–13. doi: 10.1158/0008-5472.CAN-15-1313 27013195

[2] Powles T Rosenberg JE Sonpavde GP Loriot Y Durán I Lee JL. Enfortumab vedotin in previously treated advanced urothelial carcinoma. N Engl J Med (2021) 384:1125–35. doi: 10.1056/NEJMoa2035807 PMC8450892 33577729

[3] Rosenberg J Sridhar SS Zhang J Smith D Ruether D Flaig TW. EV-101: A phase I study of single-agent enfortumab vedotin in patientswith nectin-4–positive solid tumors, including metastatic urothelial carcinoma. J Clin Oncol (2020) 38:1041–9. doi: 10.1200/JCO.19.02044 PMC7106979 32031899

[4] Fenton SE Vanderwheel DJ. Antibody-drug conjugates and predictive biomarkers in advanced urothelial carcinoma. Front Oncol (2023) 12:1069356. doi: 10.3389/fonc.2022.1069356 36686762 PMC9846350

[5] Welch JP Donaldson GA. Perforative carcinoma of colon and rectum. Ann Surg (1974) 180:734–40. doi: 10.1097/00000658-197411000-00005 PMC1343685 4423043

[6] Strati TM Sapalidis K Koimtzis GD Pavlidis E Atmatzidis S Liavas L. Sigmoido-cecal fistula: a rare case of complicated recurrent diverticulitis and a review of the literature. Am J Case Rep (2018) 19:1386–92. doi: 10.12659/AJCR.911790 PMC6266540 30464167

[7] Fukuya H Iboshi Y Wada M Sumida Y Harada N Nakamuta M. Gastric cancer presenting with ramucirumab-related gastrocolic fistula successfully managed by colonic stenting: a case report. Clin Endosc (2023). doi: 10.5946/ce.2022.117 PMC10665618 37165771

[8] Yamasaki M Daido W Funaishi K Kawamoto K Matsumoto Y Matsumoto N. Nivolumab therapy for lung cancer with tracheo-parenchymal fistula: A case report. Med (Baltim) (2018) 97:e13739. doi: 10.1097/MD.0000000000013739 PMC6320206 30558094

[9] Jung M Ryu MH Oh DY Kang M Zang DY Hwang IG. Efficacy and tolerability of ramucirumab monotherapy or in combination with paclitaxel in gastric cancer patients from the expanded access program cohort by the korean cancer study group (KCSG). Gastric Cancer (2018) 21:819–30. doi: 10.1007/s10120-018-0806-1 29427038

[10] Miyake M Miyamoto T Shimizu T Ohnishi S Fujii T Nishimura N. Tumor expression of nectin-1-4 and its clinical implication in muscle invasive bladder cancer: An intra-patient variability of nectin-4 expression. Pathol Res Pract (2022) 237:154072. doi: 10.1016/j.prp.2022.154072 35986963

[11] Kamoun A de Reyniès A Allory Y Sjödahl G Robertson AG Seiler R. A consensus molecular classification of muscle-invasive bladder cancer. Eur Urol (2020) 77:420–33. doi: 10.1016/j.eururo.2019.09.006 PMC7690647 31563503

[12] Robertson AG Kim J Al-Ahmadie H Bellmunt J Guo G Cherniack AD. Comprehensive molecular characterization of muscle-invasive bladder cancer. Cell (2017) 171:540–56.e25. doi: 10.1016/j.cell.2017.09.007 28988769 PMC5687509

[13] Chu CE Sjöström M Egusa EA Gibb EA Badura ML Zhu J. Heterogeneity in NECTIN4 expression across molecular subtypes of urothelial cancer mediates sensitivity to enfortumab vedotin. Clin Cancer Res (2021) 27:5123–30. doi: 10.1158/1078-0432.CCR-20-4175 PMC8634828 34108177

[14] Galon J Bruni D. Approaches to treat immune hot, altered and cold tumours with combination immunotherapies. Nat Rev Drug Discovery (2019) 18:197–218. doi: 10.1038/s41573-018-0007-y 30610226

[15] He Z Gu J Luan T Li H Li C Chen Z. Comprehensive analyses of a tumor-infiltrating lymphocytes-related gene signature regarding the prognosis and immunologic features for immunotherapy in bladder cancer on the basis of WGCNA. Front Immunol (2022) 13:973974. doi: 10.3389/fimmu.2022.973974 36211333 PMC9540212

[16] Helmink BA Reddy SM Gao J Zhang S Basar R Thakur R. B cells and tertiary lymphoid structures promote immunotherapy response. Nature (2020) 577:549–55. doi: 10.1038/s41586-019-1922-8 PMC8762581 31942075

[17] Petitprez F de Reyniès A Keung EZ Chen TW Sun CM Calderaro J. B cells are associated with survival and immunotherapy response in sarcoma. Nature (2020) 577:556–60. doi: 10.1038/s41586-019-1906-8 31942077

[18] Cabrita R Lauss M Sanna A Donia M Larsen MS Mitra S. Tertiary lymphoid structures improve immunotherapy and survival in melanoma [published correction appears in Nature. Nature (2020) 577:561–5. doi: 10.1038/s41586-019-1914-8 31942071

[19] Yang L Li A Liu F Zhao Q Ji S Zhu W. Immune profiling reveals molecular classification and characteristic in urothelial bladder cancer. Front Cell Dev Biol (2021) 9:596484. doi: 10.3389/fcell.2021.596484 33777927 PMC7990773

[20] Adib E Nassar AH Akl EW Abou Alaiwi S Nuzzo PV Mouhieddine TH. CDKN2A alterations and response to immunotherapy in solid tumors. Clin Cancer Res (2021) 27:4025–35. doi: 10.1158/1078-0432.CCR-21-0575 PMC8900067 34074656

[21] Basin MF Bratslavsky G Nahhas N Basnet A Goldberg H Necchi A. Novel synthetic lethality drug target in urothelial bladder cancer based on MTAP genomic loss. Urol Oncol (2023) 41:109.e15–109.e22. doi: 10.1016/j.urolonc.2022.10.001 36443178

