# Supplementary figures and images for: Metastatic bladder cancer forming a sigmoidorectal fistula after enfortumab vedotin therapy: a case report
Source: Front Oncol. 2023 Nov 9;13:1274494. doi: 10.3389/fonc.2023.1274494 (PMC10665906; doi:10.3389/fonc.2023.1274494)

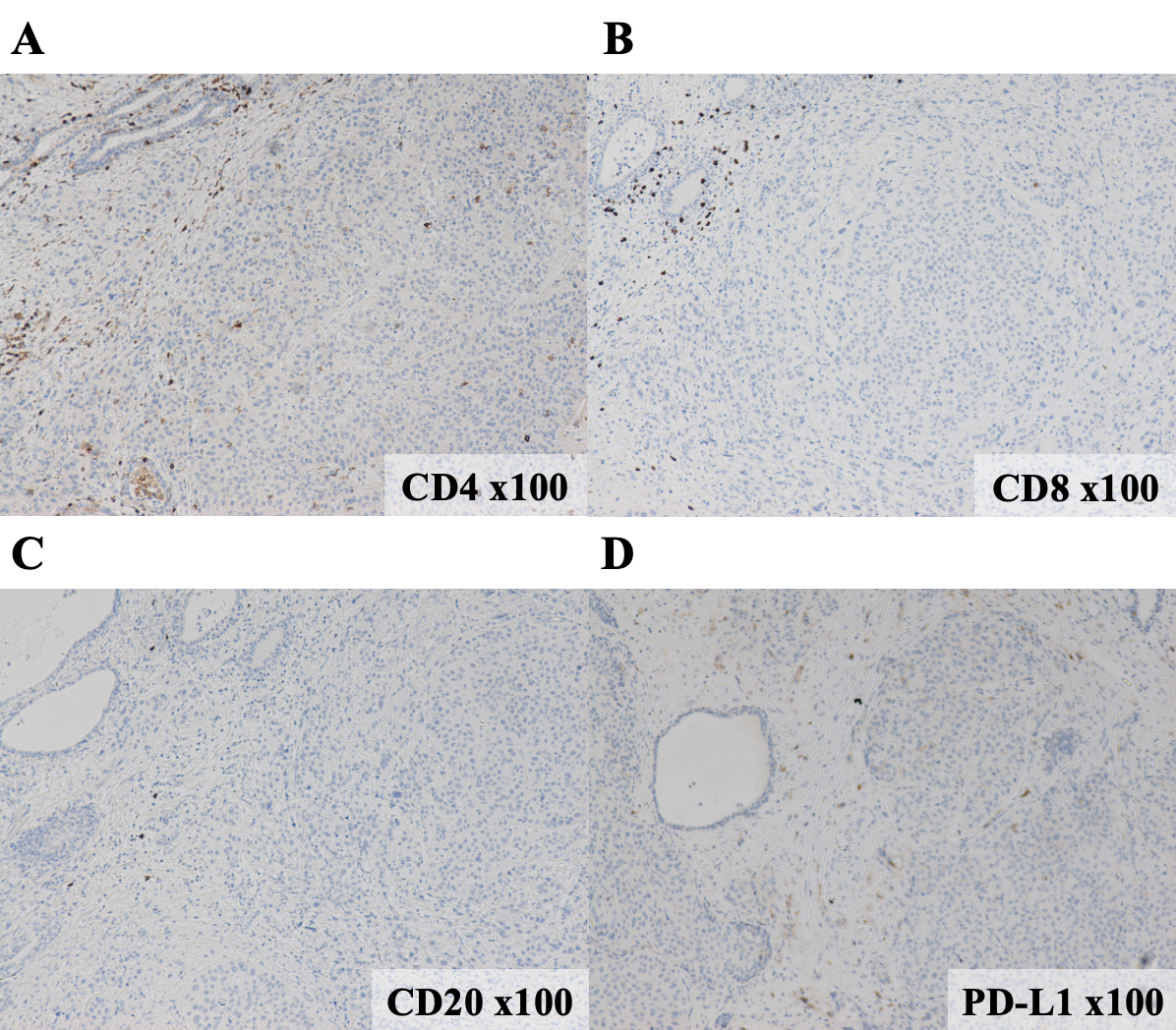

Supplement: Supplementary file 1 [file Image_1.jpeg]
